# A drug‐selectable acoustic reporter gene system for human cell ultrasound imaging

**DOI:** 10.1002/btm2.10584

**Published:** 2023-08-02

**Authors:** Alessandro R. Howells, Phoebe J. Welch, John Kim, Craig R. Forest, Chengzhi Shi, Xiaojun Lance Lian

**Affiliations:** ^1^ Department of Biomedical Engineering Pennsylvania State University Pennsylvania USA; ^2^ George W. Woodruff School of Mechanical Engineering Georgia Institute of Technology Atlanta Georgia USA; ^3^ Parker H. Petit Institute for Bioengineering and Bioscience, Georgia Institute of Technology Atlanta Georgia USA; ^4^ Huck Institutes of the Life Sciences, Pennsylvania State University Pennsylvania USA; ^5^ Department of Biology Pennsylvania State University Pennsylvania USA

**Keywords:** acoustic reporter genes, cell engineering, ultrasound imaging

## Abstract

A promising new field of genetically encoded ultrasound contrast agents in the form of gas vesicles has recently emerged, which could extend the specificity of medical ultrasound imaging. However, given the delicate genetic nature of how these genes are integrated and expressed, current methods of producing gas vesicle‐expressing mammalian cell lines requires significant cell processing time to establish a clonal/polyclonal line that robustly expresses the gas vesicles sufficiently enough for ultrasound contrast. Here, we describe an inducible and drug‐selectable acoustic reporter gene system that can enable gas vesicle expression in mammalian cell lines, which we demonstrate using HEK293T cells. Our drug‐selectable construct design increases the stability and proportion of cells that successfully integrate all plasmids into their genome, thus reducing the amount of cell processing time required. Additionally, we demonstrate that our drug‐selectable strategy forgoes the need for single‐cell cloning and fluorescence‐activated cell sorting, and that a drug‐selected mixed population is sufficient to generate robust ultrasound contrast. Successful gas vesicle expression was optically and ultrasonically verified, with cells expressing gas vesicles exhibiting an 80% greater signal‐to‐noise ratio compared to negative controls and a 500% greater signal‐to‐noise ratio compared to wild‐type HEK293T cells. This technology presents a new reporter gene paradigm by which ultrasound can be harnessed to visualize specific cell types for applications including cellular reporting and cell therapies.

**Translational Impact Statement**

Our new inducible, drug‐selectable acoustic reporter gene technology significantly streamlines the production of mammalian cells expressing gas vesicles for ultrasound contrast. This facilitates the visualization of specific cell types and opens up new avenues in cellular reporting and therapy. Our breakthrough method avoids the need for laborious cell processing, and delivers robust contrast. This new drug‐selectable acoustic reporter gene technology could accelerate progress in medical ultrasound imaging research and potentially enhancing patient care.

## 1 INTRODUCTION

The use of reporter genes to identify certain cell populations has become a ubiquitous laboratory technique, spanning many aspects of biological sciences. The most common demonstration of reporter genes in biological sciences are fluorescent proteins, which can be integrated into the cell for endogenous fluorescence labeling.^1^, ^2^, ^3^, ^4^ Hundreds of fluorophores that span nearly the entire visible spectrum have been discovered and optimized to fluoresce in response to photon absorption.^5^, ^6^ However, due to the scattering nature of tissue in the visible electromagnetic spectrum, imaging such fluorescent reporters in vivo is limited to several millimeters of depth in tissue or requires transparent animal models.^7^, ^8^ Alternatively, researchers utilize histology for observing tissue composition throughout the entire region of interest, but this requires animal euthanasia for tissue sectioning or biopsy, thus eliminating the opportunity for long‐term in vivo monitoring of cell populations within the same animal.^9^, ^10^

Noninvasive imaging techniques, such as ultrasound, magnetic resonance imaging (MRI), and positron emission tomography (PET), exist in healthcare as tools to image tissue structures in vivo for clinical diagnostics.^11^ Notably, ultrasound is a useful imaging modality for various applications as it does not require any harmful ionizing radiation and can be achieved using relatively inexpensive and transportable equipment.^12^, ^13^, ^14^, ^15^ Ultrasound contrast agents can be used, such as the Food and Drug Administration‐approved lipid or protein‐shelled microbubbles, to improve the contrast of in vivo ultrasound imaging.^16^, ^17^, ^18^, ^19^ Newer ultrasound contrast agents, including nanobubbles and perfluorocarbon nanodroplets, are also being investigated for a variety of biomedical applications, including tumor extravasation and targeted drug delivery.^20^, ^21^, ^22^, ^23^, ^24^, ^25^, ^26^, ^27^ However, these newer nanodroplet and nanobubble contrast agents are still limited in their use cases as they function quite similarly to microbubbles, have limited stability in vivo, are not genetically encodable, and cannot currently be used to image precise locations of cells in deep tissue due to the small quantity of droplets that are taken up by cells.^27^, ^28^, ^29^ Ideally, an ultrasound contrast agent would be developed that could be genetically encoded into cells and used as an acoustic analog to fluorescent reporter genes.

Work in the field of genetically‐encodable ultrasound contrast agents has turned to gas vesicles (GVs), which many bacteria and archaea express to make these organisms buoyant in aqueous environments.^30^ These nanoscale GVs are comprised of gas vesicle proteins (Gvps), which self‐assemble to form a gas‐filled space within the cytoplasm. Initially, GVs isolated from bacteria were shown to be a useful ultrasound contrast agent, both in vitro and in vivo, by using ultrasound pulses with sufficient pressure to induce GV collapse, causing a sudden loss in ultrasound contrast in the targeted region, or by using unique ultrasound wave patterns to isolate the nonlinear scattering of the GVs from the surrounding environment.^31^, ^32^, ^33^, ^34^, ^35^ More recently, genes encoding a specific combination of Gvps have been successfully expressed within mammalian cell lines and form GVs, termed mammalian acoustic reporter genes (mARG).^36^ These mARGs integrate into the mammalian cell genome, and after 3 days of induced expression, the GVs generated by the cell provide ultrasound contrast sufficient to serve as a reporter of these cells. Furthermore, these cells can re‐express the GVs several days postcollapse.^36^

Nevertheless, numerous challenges arise when developing new mARG cell lines. For one, isolating the cells that have been properly transfected via random integration requires fluorescence‐activated cell sorting (FACS) followed by single‐cell cloning, a costly, inefficient, and time‐consuming process. Additionally, current mARG constructs only enable inducible GV expression in Tet‐On cell lines, limiting the application of this technology to only previously engineered Tet‐On cells.^36^ For the advancement of mARG technology, it's crucial to develop new drug‐selectable mARG constructs. This development would simplify the process of isolating cells that robustly express GVs.

Here, we present our mammalian acoustic reporter gene system that aims to improve the accessibility and efficiency of this technology. Accessibility is improved by incorporating the Tet‐On 3G gene into our constructs, thus enabling application of the doxycycline‐inducible mARG technology into wild‐type cells rather than only transgenic Tet‐On cells. To address the efficiency bottleneck caused by single‐cell cloning and FACS, a drug‐selectable strategy is implemented using several common antibiotics. Our system is labeled mARG_ds_ for drug‐selectable mammalian acoustic reporter genes. The utility of our new mARG_ds_ construct design is demonstrated within HEK293T cells by optically and ultrasonically validating GV expression.

## 2 RESULTS

### 2.1 Drug selectable mARG design (mARG_ds_ )

**FIGURE 1:**
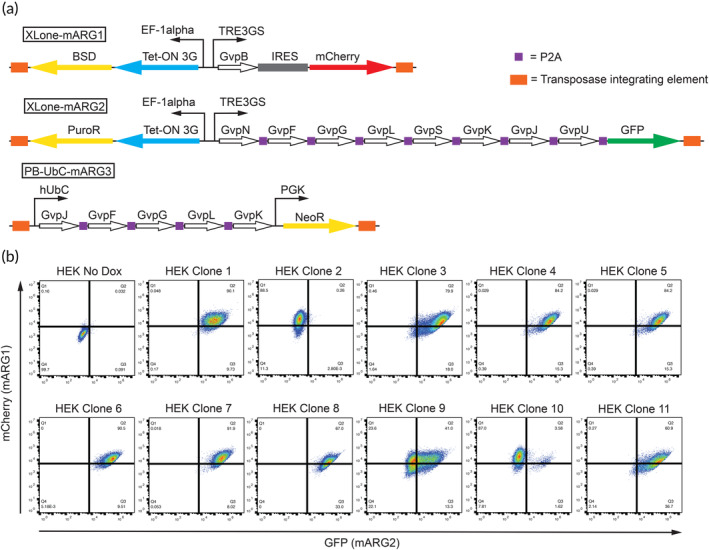
Design of mARG_ds_ HEK293T clones. (a) Schematic of the transposase integrating mARG_ds_ construct designs. From left to right, the XLone‐mARG1 cassette possesses the Blasticidin resistant gene and the Tet‐On 3G system downstream of the constitutive EF‐1α promoter, and the mARG GvpB gene and mCherry downstream of the Dox inducible TRE3GS promoter. The XLone‐mARG2 cassette possesses the Puromycin resistant gene and the Tet‐On 3G system downstream of the constitutive EF‐1α promoter, and the mARG GvpF, GvpG, GvpL, GvpS, GvpK, GvpJ, GvpU genes (separated by P2A) and GFP downstream of the Dox inducible TRE3GS promoter. Finally, PB‐UbC‐mARG3 cassette possesses the mARG GvpF, GvpG, GvpL, and GvpK genes downstream of the constitutive hUbC promoter and Geneticin resistant gene downstream of the constitutive PGK promoter. (b) Flow cytometry of mARG_ds_ HEK293T clonal lines against GFP (XLone‐mARG2) and mCherry (XLone‐mARG1).

We aimed to induce GV expression in human cells following a similar approach to the Shapiro group,^36^ with two important differences. First, whereas the Shapiro group relies on transgenic Tet‐On cells for their mARG constructs, our newly developed mARG_ds_ constructs allow for doxycycline‐inducible GV expression in non‐Tet‐On 3G cells. This eliminates the need for Tet‐On 3G engineered cell lines. For the second modification, we noted in our first attempt to replicate Shapiro's work that GV formation was very low in our HEK293T clonal lines as no ultrasound signal was generated.^36^ To address this issue, streamline the workflow, and increase the efficiency of producing robust mARG‐expressing clonal HEK293T lines, each of the three mARG cassettes was made drug selectable against its own unique antibiotic (Figure 1a).

### 2.2 Development of mARG_ds_ HEK293T clonal lines

**FIGURE 2:**
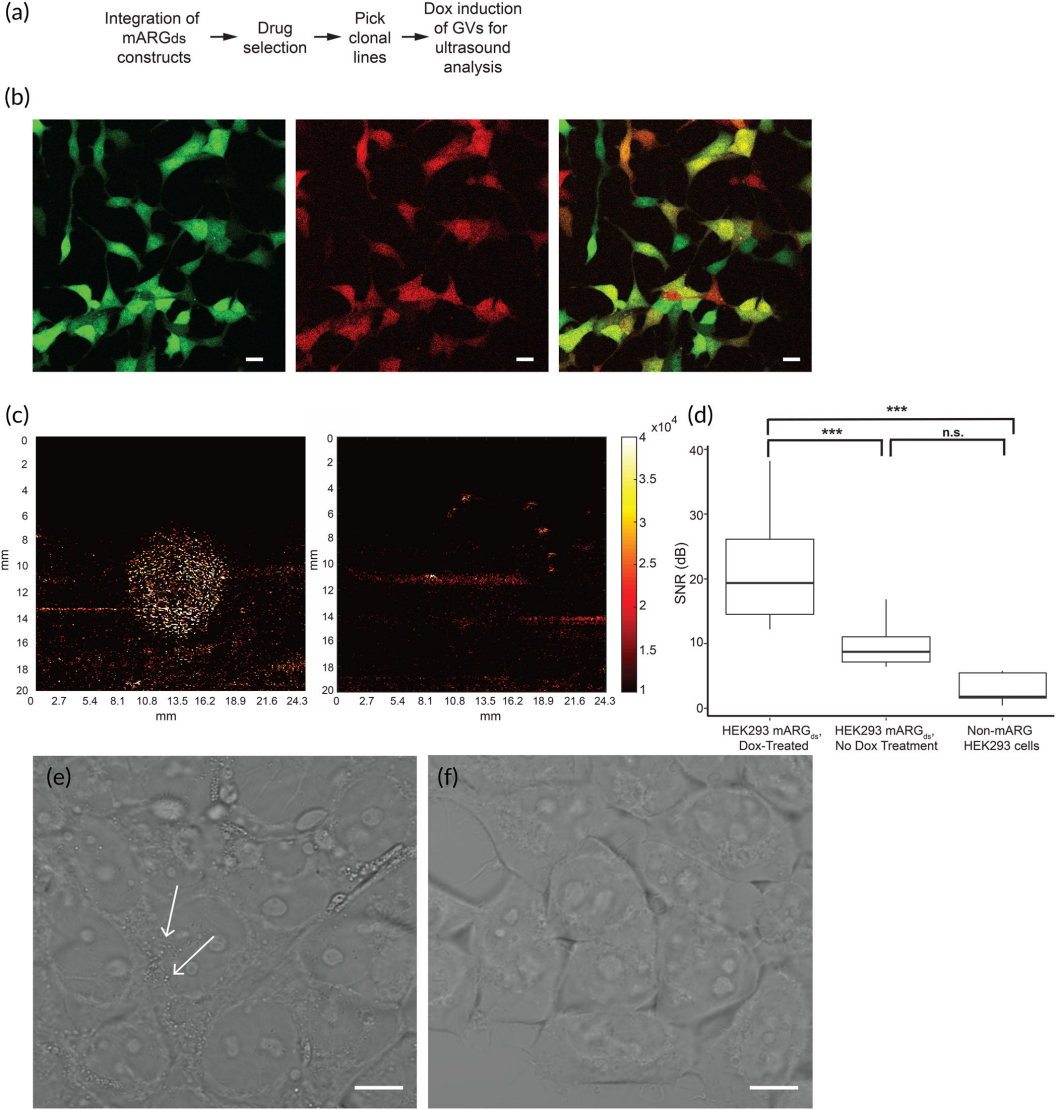
Single cell mARG_ds_ HEK293T cells express gas vesicles and produce ultrasound contrast. (a) Process flow for generating gas vesicle‐producing single‐cell clone HEK293T cell lines. (b) Clone 6 HEK293T mARG_ds_ gene expression validated by fluorescence microscopy. Scale bar = 10 μm. (c) Ultrasound contrast is produced by doxycycline‐treated HEK293T mARG_ds_ single cell clones after 3 days of treatment (left panel) whereas cells not treated with doxycycline produce no ultrasound contrast (right panel). (d) Signal to noise ratio of ultrasound imaging is significantly stronger in doxycycline‐treated cells compared to non‐doxycycline treated cells and non‐mARG_ds_ cells, indicative of gas vesicle production (*n* = 6). (e) Phase contrast image of HEK293T mARG_ds_ cells after 3 days of doxycycline treatment with small puncta spread throughout the cell (white arrows), indicative of gas vesicle presence. (f) Phase contrast image of HEK293T mARG_ds_ cells undergoing no doxycycline treatment.

The mARG_ds_ plasmids were introduced into HEK293T cells via transfection, in conjunction with a plasmid that encodes for the transposase enzyme. Following the transfection, the cell culture media was progressively supplemented with incremental concentrations of three antibiotics—geneticin (G418), blasticidin (BSD), and puromycin (Puro). This step was performed to isolate cells expressing GVs. This mARG_ds_ HEK293T population was then single cell plated and 11 clonal lines were picked and expanded. These 11 clonal lines were treated with 5 μg/mL Dox and 5 mM sodium butyrate (to prevent epigenetic silencing) for 72 h in the presence of the 3‐antibiotic cocktail and assessed for green fluorescence protein (GFP) and mCherry expression via flow cytometry (PB‐UbC‐mARG3 cassette does not possess a reporter fluorophore). Based on this, five clones (Figure 1b) yielded the highest double positivity and were the focus of further characterization. These clones were expanded in 6‐well plates until 90%–100% confluent and integrated into ultrasound experiments after doxycycline and sodium butyrate treatment for 72 h (Figure 2a).

Using fluorescence microscopy, it was observed that clone 6 produced a high percentage of double‐positive fluorescent cells (Figure 2b). This clonal line also yielded strong ultrasound contrast in the B mode imaging based on measuring the signal‐to‐noise ratio (SNR) of the cell sample compared to the background noise and control groups, which included clone 6 cells cultured without doxycycline and sodium butyrate and unmodified HEK293T cell lines (Figure 2c,d). The average SNR from doxycycline‐treated cells was 21.75 dB (125% greater than non‐doxycycline treated mARG_ds_ HEK293T cells) with peak values reaching 38.2 dB. Additionally, clusters of GVs were observed in the doxycycline treated cells under phase contrast microscopy, whereas none were observed in the non‐doxycycline treated mARG_ds_ cells (Figure 2e,f). Additionally, after prolonged culturing in the drug selection media, this clone still exhibited these key features (Figure S1). These results demonstrate that inclusion of the Tet‐On 3G gene within the mARG cassette enables doxycycline induced GV expression, when starting with wild‐type (non‐Tet‐On) HEK293T cells and single‐cell cloning.

### 2.3 Development of mARG_ds_ HEK293T mixed population cell line

**FIGURE 3:**
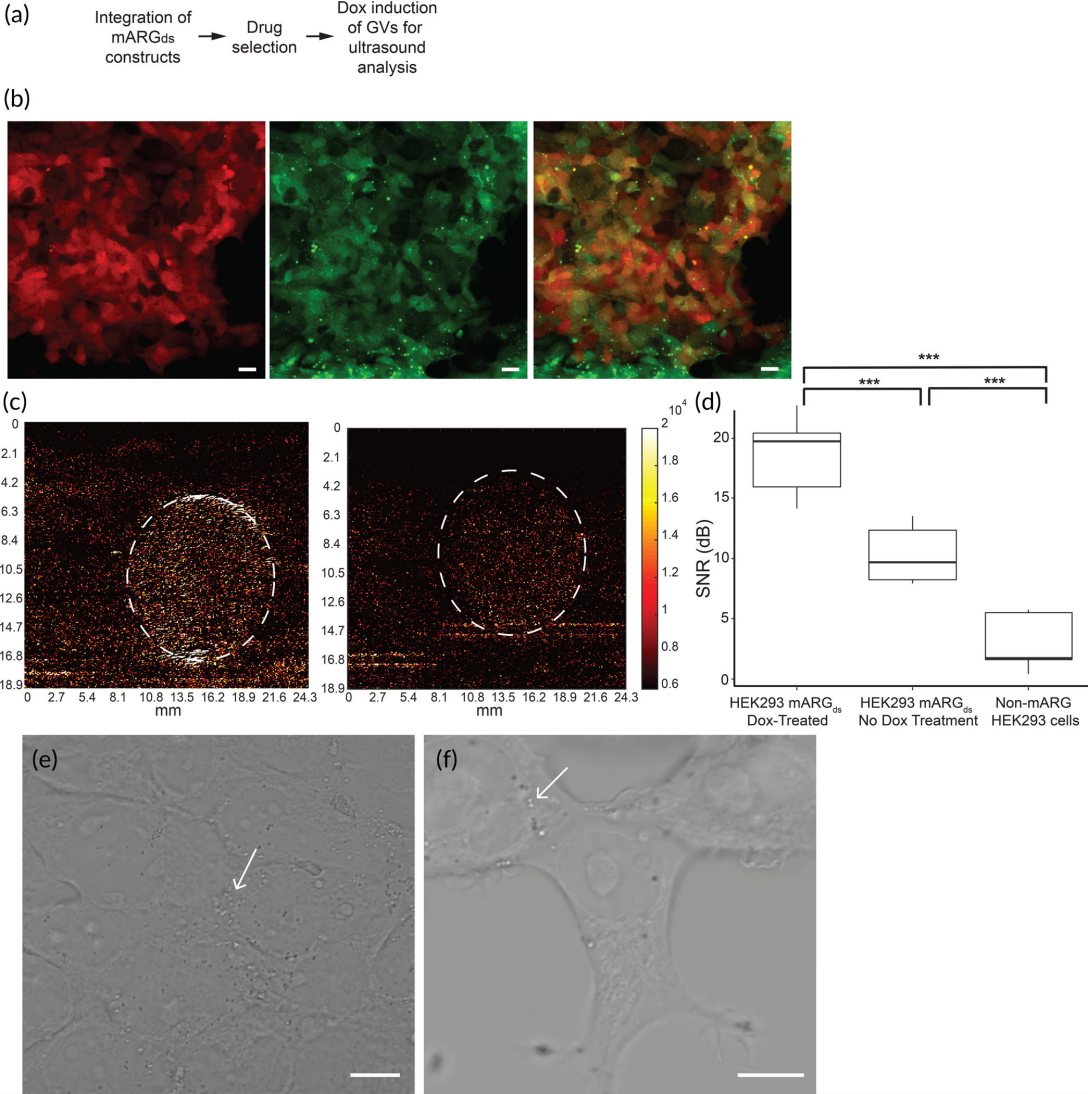
Mixed population HEK293T mARG_ds_ cells produce sufficient ultrasound contrast after several days of antibiotic selection and doxycycline treatment. (a) Process flow for generating gas vesicle‐producing mixed population HEK293T cells. (b) Antibiotic selected and doxycycline‐treated HEK293T mARG_ds_ mixed population cells properly integrated the mARG_ds_ genes. Scale bar = 10 μm. (c) Doxycycline‐treated HEK293T mARG_ds_ mixed population cells produce stronger ultrasound contrast (left panel) compared to non‐doxycycline treated mARG_ds_ mixed population cells. (d) mARG_ds_ mixed population HEK293T cells exhibit a stronger signal to noise (SNR) ratio after 3 days of doxycycline treatment compared to non‐doxycycline treated mARG_ds_ cells and non‐mARG cells (*n* = 7). (e) Phase contrast image of HEK293T mARG_ds_ cells after 24 h of doxycycline treatment with small puncta spread throughout the cell (white arrows), indicative of gas vesicle presence. (f) Phase contrast image of HEK293T mARG_ds_ cells undergoing no doxycycline treatment. Small puncta are still present (white arrows), though in much smaller quantities than doxycycline‐treated cells.

The next step in streamlining GV‐expression in human cells was to determine whether the drug‐selectable strategy alone was sufficient to generate a robust enough mARG_ds_ mixed cell population that would not require single‐cell cloning (Figure 3a). The drug‐selected mixed population, that is, the initial cells used for the single cell plating and cloning, were treated with 5 μg/mL doxycycline and 5 mM sodium butyrate for 72 h to induce GV expression. Cells were optically validated for the expression of the mARG_ds_ genes after just 24 h of doxycycline treatment (Figure 3b). When ultrasonically imaged after 72 h of treatment, the doxycycline‐treated cells exhibited significantly stronger ultrasound contrast than non‐doxycycline‐treated mARG_ds_ cells as evidenced by SNR calculations (Figure 3c,d). The average SNR of the doxycycline‐treated cells was 18.5 dB, 80% greater than the SNR of non‐doxycycline‐treated cells and 500% stronger than the HEK293T cells. Additionally, GV expression was optically observed using phase contrast imaging after 24 h of doxycycline treatment (Figure 3e). Interestingly, the ultrasound contrast of non‐doxycycline treated mARG_ds_ mixed population cells was higher than that of non‐mARG_ds_ HEK293T cells, and trace GVs were observed in optical images of the mARG_ds_ cells that were not treated with doxycycline (Figure 3f). Possible reasons for the presence of GVs in these cells might include the leaky expression of the Gvps, even when cultured in Tet‐approved media. Additionally, the observed phenomena could be attributed to our use of a mixed‐cell population. Regardless, this trace GV expression still resulted in a significantly lower SNR than that from the doxycycline‐treated mARG_ds_ cells.

After prolonged culturing in the drug selection media, our drug‐selected polyclonal line still exhibited a higher SNR, of 29 dB after doxycycline treatment, compared to a SNR of 6.6 dB, of the same cells not treated with doxycycline (Figure S2). The doxycycline‐treated cells were also still highly GFP and mCherry double positive and GV formation was confirmed via phase contrast microscopy (Figure S2). This highlights how maintaining the mARG_ds_ HEK293T for a prolonged period in the antibiotic cocktail enables stable GV formation and theoretically prevents silencing of the three randomly transposase inserted mARG_ds_ cassettes. Additionally, based on these results, our drug‐selectable strategy is indeed sufficient enough to generate a polyclonal cell line that can robustly express mARG_ds_, eliminating the need for FACS or single‐cell cloning.

## 3 DISCUSSION

Here, we demonstrate the expression of drug‐selectable mARGs, labeled mARG_ds_, within HEK293T cells. Our strategy incorporates the Tet‐On 3G system, enabling doxycycline‐inducible expression of the mARG_ds_ into HEK293T cells. As the mARG design stands from the Shapiro group, drug‐inducible mARG integration into mammalian cells is relegated to cell lines that already have the Tet‐On 3G system integrated into their genome. Our strategy broadens its application potential without the need to genetically integrate the Tet‐On 3G system prior. Additionally, our mARG_ds_ construct design incorporates unique drug‐resistant genes downstream of each of the three mARG cassettes. By culturing these cells in the antibiotic cocktail, it enables us to purify cells that have successfully integrated all three mARG_ds_ cassettes, forgoing the need for FACS or single‐cell cloning.

The mARG_ds_ system builds upon the pioneering work of the Shapiro group, who originally developed the mARG constructs. Their research demonstrated the successful integration of GV genes from bacterial strains into mammalian cells, specifically HEK293T and Chinese hamster ovary cells (CHO‐K1). They also engineered mammalian cell lines with doxycycline‐inducible GV expression using transformed cell lines, particularly HEK293 Tet‐On cells, which express the transactivator Tet‐On 3G gene. However, the generation of these cells had a notably low throughput, requiring the use of techniques like FACS and single‐cell cloning to identify triple‐positive fluorescent cells and determine which ones produced GVs after doxycycline treatment.

To enhance throughput without relying on various cell separation techniques, we introduced drug‐selectable elements into each of the three mARG constructs, making them selectable against unique antibiotics. Furthermore, we directly integrated the Tet‐On 3G gene into the mARG construct, enabling the induction of GV expression by doxycycline in wild‐type cells. For the integration of these mARG_ds_ plasmids, we chose to employ HEK293T cells because they are highly suitable for transfection and commonly utilized in various cell engineering applications.

Thus far, mARG and mARG_ds_ expression has been demonstrated in mammalian and human cell lines that are predisposed toward easy transfection, including the HEK293T cells used in this work as well as HEK293 and CHO‐K1 cells in Farhadi et al. HEK293 and CHO cells are widely used for experiments involving transfection due to their high transfection efficiency. However, numerous factors play a role in whether transfection can successfully be implemented in a cell line, including the cell confluency, cell passage number, and the type and amount of transfection agent used.

Due to the delicate nature of how each Gvp must be expressed in a narrow stoichiometric ratio for them to self‐assemble to correctly form the GVs, the efficiency at which you can generate a mARG clonal lines that robustly expresses many GVs is extremely low. Based on Farhadi et al., 6/30 HEK clonal lines generated greater than 1 GV per cell, and as reported, 1 of these 30 clonal lines generated about 45 GVs per cell.^16^ Every nuanced attempt at further increasing the efficiency by which researchers can generate these cells that have integrated all three mARG constructs is essential for furthering the application of mARGs.

## 4 CONCLUSIONS

We developed a new series of mammalian acoustic reporter constructs that are drug selectable to improve the success rate of GV expression. These mARG_ds_ plasmids also have the Tet‐On system integrated in the plasmid backbone, which means doxycycline‐inducible GV expression can be implemented in wild‐type (not constitutively expressing Tet‐On) cells. In this study, we integrated our mARG_ds_ design in wild‐type HEK293T cells and prevented gene silencing of all three cassettes by continually culturing them in the presence of the drug cocktail. The mARG_ds_ genes streamlined the workflow for developing mARG_ds_ clonal HEK293T lines by eliminating the need for FACS‐mediated purification of cell populations that had successfully integrated all three cassettes, as evidenced by ultrasonic and optical imaging. Finally, we demonstrate that our mARG_ds_ construct strategy does not require the establishment of mARG clonal lines, and a drug‐selected mixed population of cells is sufficient for ultrasound contrast generation. The findings detailed in this manuscript indicate that doxycycline‐inducible GV expression can be applied to non‐Tet‐On HEK293T cells, rendering these acoustic reporter genes useful for various applications such as cell transplantation and tissue grafting.

## 5 MATERIALS AND METHODS

### 5.1 Maintenance of HEK293T cells

HEK293T cells were selected for these experiments due to their ease of maintenance and transfection. HEK293T cells (System Biosciences) were cultured in DMEM + 10% FBS (ThermoFisher) on uncoated Corning Falcon 6 well plates (Corning). Complete media changes were performed daily by prewarming the DMEM + 10% FBS to 37°C prior to aspirating and replacing old media. Passages were conducted when cells reached about 80% confluency by suspending the cells using 0.25% Trypsin‐EDTA (ThermoFisher), centrifuging for 4 min at 1000 rpm, and sparsely replating in fresh media. Cell line stocks were frozen in DMEM + 10% DMSO + 10% FBS.

### 5.2 Drug selectable mARG plasmid cloning

DNA fragments of mARG1‐1, mARG1‐2, and mARG1‐3 (Addgene # 134343, 134344, 134345, respectively) were amplified using primers (Table S1) obtained from the In‐Fusion Cloning Primer Design Tool (Takara Bio USA) and Q5 High‐Fidelity DNA polymerase (New England Biolabs) according to the manufacturer's instructions. These PCR fragments were then cloned into the drug‐selectable XLone‐GFP (Addgene #96930), XLone‐Puro eGFP (Addgene #140027), and PB‐UbC‐GCaMP6f‐polyA‐PGK‐Neo (Addgene #160049) plasmid backbones, respectively, using the In‐Fusion® HD Cloning Kit (Takara Bio USA, Inc.), according to the manufacturer's protocol. Each newly cloned mARG plasmid cassette now possesses its own unique drug‐resistant gene, XLone‐mARG1‐Bsd‐mCherry (Blasticidin selectable, mCherry reporter), XLone‐mARG2‐puro‐GFP (Puromycin selectable, GFP reporter), and PB‐UBC‐mARG3‐neo (Geneticin selectable) (Addgene # 173798, 173792, and 173793, respectively), hence the ds subscript on our mARG_ds_ cassettes (Figure 1a).

### 5.3 Development of drug‐selectable mARG HEK293T clonal and mixed population lines

After plasmid cloning, these 3 new plasmids were transfected into HEK293T cells via lipofection with TransIT‐LT1 Transfection Reagent (Mirus Bio) at a 2.5:1 molar ratio with the EF1a‐hyPBase transposase plasmid, according to manufacturer's instructions. At this point, the HEK293T cells were cultured in DMEM supplemented with Tet‐approved FBS to prevent premature expression of the GV‐producing plasmids. Cells were selected with 50 μg/mL G418, 5 μg/mL BSD, and 0.5 μg/mL Puro for 3 days followed by another 4 days with 200 μg/mL G418, 20 μg/mL BSD, and 2 μg/mL Puro. In total, a 7‐day drug selection was needed. These cells were used for mixed population analysis and single‐cell cloning.

Cells were single‐cell cloned by sparsely passaging onto a 10 cm petri dish. Selected clones were manually picked and propagated. After a 72‐h treatment with 5 μg/mL doxycycline and 5 mM sodium butyrate (added to prevent epigenetic silencing of the mARGds construct), these clonal lines were analyzed for mCherry and GFP expression using fluorescent microscopy or flow cytometry.

### 5.4 Flow cytometry

After 72‐h treatment with 5 ug/mL doxycycline, sodium butyrate, and drug selection media, cells were resuspended using the resuspension media described above. Negative controls did not receive doxycycline treatment. After centrifugation, cell pellets were resuspended in 800 μL of FlowBuffer‐1 (PBS + 0.5% BSA) and pipetted into a cytometer flow tube. Flow for mCherry and GFP expression were performed using a BD Accuri C6 Plus flow cytometer (BD), using the PerPC‐A and FITC‐A channels, respectively. Raw data were then processed using the Flowjo software (Figure 1b).

### 5.5 Ultrasound imaging and data analysis

On the day of experiments, cells were trypsinized, counted using a hemocytometer, and suspended in liquid, low‐gelling temperature agarose (1%, ThermoFisher) at a concentration of approximately 20 million cells/mL. This agarose‐cell suspension was loaded into sample holders comprised of a thin‐walled plastic tube attached to a 1 mL syringe, which could then be tethered to a 3D‐printed syringe holder. This created a circular cross‐section of cells that was easy to focus the ultrasound transducers on and localize any GV collapse during experiments. These cell samples were lowered into a 37°C heated water bath filled with degassed, deionized water. A single‐element focused ultrasound transducer operating at 1.05 MHz (H‐101, SonicConcepts) was fitted with a coupling cone backfilled with degassed, deionized water and focused such that the tip of the coupling cone (the focal spot of the ultrasound transducer) was aligned with the cell sample holder. This transducer sent short 10‐cycle bursts of focused ultrasound with pressures ranging from 2 to 4 MPa peak‐negative pressure. These pressures are sufficiently high to guarantee GV collapse in the cells. Lower pressures may be used as described in previous literature.^37^ Orthogonal to the cell sample holder was an L22‐8v CMUT linear array transducer (Kolo Medical) used to capture images of the cells and any GV collapse. This multiplexing transducer operated at its center frequency of 15.625 MHz and operated at ~300 frames per second. RF data from the linear array transducer was collected using a Vantage 256 ultrasound imaging system (Verasonics) and reconstructed later as B‐mode images for analysis. The linear‐array transducer and single‐element transducer were synchronized such that the single‐element transducer transmitted starting on specific frames numbers so that it was easy to later identify when the GV collapse occurred. Twenty‐five precollapse frames and 100 postcollapse frames were captured per 10‐cycle burst sine wave event.

GV collapse was quantified by calculating the SNR of the cells compared to the background noise surrounding the sample in B‐mode ultrasound images. Differential B‐mode images were created by subtracting postcollapse ultrasound frames from previous frames. A custom Matlab script (MathWorks) was created that calculated the signal within the circular confines of the agarose sample holder and compared it to the noise of the surrounding environment in the water bath. A circular region of interest (ROI) was drawn over the sample holder and the signal was calculated by summing and averaging all of the pixel intensities within that circular ROI. For the noise, a circular ROI was created adjacent to the sample holder and the averaged sum of that region's pixel intensities constituted the noise value. The formula used to calculate the signal‐to‐noise ratio (SNR) is $\mathrm{SNR}\;\left(\mathrm{dB}\right)=20*\log\left(\frac{P_{\text{signal}}}{P_{\text{noise}}}\right)$, where $P_{\text{Signal}}=\frac{1}{\mathrm{MN}}\sum_{i=1}^{M}\sum_{j=1}^{N}A_{\text{Signal}}^{2}\;\left(i,j\right)$ and $P_{\text{noise}}=\frac{1}{\mathrm{MN}}\sum_{i=1}^{M}\sum_{j=1}^{N}A_{\text{noise}}^{2}\;\left(i,j\right)$.

These SNR values were used to determine sufficient ultrasound contrast produced by the collapsing GVs compared to negative controls. Box plots and statistical analysis were conducted in R Studio. One‐way ANOVA and Tukey's *t*‐test were used to determine statistical significance in variance between cell groups.

### 5.6 Phase contrast and fluorescence imaging

Optical images were captured using Zeiss Laser Scanning Microscopes (700 and 900 Confocal, Zeiss) in the Optical Microscope Core in the Georgia Institute of Technology. HEK293T mARG_ds_ cells were cultured on glass‐bottomed petri dishes and treated with doxycycline for at least 24 h before imaging occurred. To capture images of the GVs, the microscope was configured to capture phase contrast images at 40× magnification. Fluorescent images were captured using 488 nm and 555 nm lasers to excite GFP and mCherry, respectively.

## AUTHOR CONTRIBUTIONS

*Conceptualization*: Chengzhi Shi and Xiaojun Lance Lian. *Methodology*: Alessandro R. Howells and Phoebe J. Welch. *Investigation*: Alessandro R. Howells, Phoebe J. Welch, and John Kim. *Visualization*: Alessandro R. Howells, Phoebe J. Welch, and John Kim. *Writing—original draft*: Phoebe J. Welch and Alessandro R. Howells. *Writing—review and editing*: Phoebe J. Welch, Alessandro R. Howells, John Kim, Chengzhi Shi, and Xiaojun Lance Lian. *Supervision*: Chengzhi Shi, Xiaojun Lance Lian, and Craig R. Forest.

## FUNDING INFORMATION

Phoebe J. Welch, John Kim, and Chengzhi Shi are supported by the National Science Foundation under the grants CMMI‐2142555 and ECCS‐2102129. Alessandro R. Howells and Xiaojun Lance Lian are supported by the National Science Foundation under the grant CBET‐1943696.

## CONFLICT OF INTEREST STATEMENT

The authors declare no conflict of interest.

### PEER REVIEW

The peer review history for this article is available at https://www.webofscience.com/api/gateway/wos/peer-review/10.1002/btm2.10584.

## Supporting information


**Figure S1.** HEK293T mARG_ds_ clonal cell line after freezing, thawing, and passaging for 5 passages post‐thaw. Cells exhibit ultrasound contrast after doxycycline treatment (SNR = 24 dB, A) compared to cells that underwent no doxycycline treatment (SNR = 5.5 dB, B). Doxycycline‐treated cells exhibit double‐positive fluorescence (C) and evidence of gas vesicle formation under phase‐contrast imaging (D) compared to negative controls (E). Scale bar = 10 μm.
**Figure S2.** HEK293T mARG_ds_ mixed‐population cell line after freezing, thawing, and passaging for 5 passages post‐thaw. Cells exhibit ultrasound contrast after doxycycline treatment (SNR = 29 dB, A) compared to cells that underwent no doxycycline treatment (SNR = 6.6 dB, B). Doxycycline‐treated cells exhibit double‐positive fluorescence (C) and evidence of gas vesicle formation under phase‐contrast imaging (D) compared to negative controls (E). Scale bar = 10 μm.

**Table S1.** Primers used to PCR amplify mARG cassettes in order to clone into their respective drug‐resistant backbone via In‐Fusion cloning.

## Data Availability

The data that support the findings of this study are available from the corresponding author upon reasonable request.
