# Supplementary material for: A drug‐selectable acoustic reporter gene system for human cell ultrasound imaging
Source: Bioeng Transl Med. 2023 Aug 2;9(2):e10584. doi: 10.1002/btm2.10584 (PMC10905554; doi:10.1002/btm2.10584)
Supplement: Supplementary file 1 — Figure S1. HEK293T mARGds clonal cell line after freezing, thawing, and passaging for 5 passages post‐thaw. Cells exhibit ultrasound contrast after doxycycline treatment (SNR = 24 dB, A) compared to cells that underwent no doxycycline treatment (SNR = 5.5 dB, B). Doxycycline‐treated cells exhibit double‐positive fluorescence (C) and evidence of gas vesicle formation under phase‐contrast imaging (D) compared to negative controls (E). Scale bar = 10 μm. Figure S2. HEK293T mARGds mixed‐population cell line after freezing, thawing, and passaging for 5 passages post‐thaw. Cells exhibit ultrasound contrast after doxycycline treatment (SNR = 29 dB, A) compared to cells that underwent no doxycycline treatment (SNR = 6.6 dB, B). Doxycycline‐treated cells exhibit double‐positive fluorescence (C) and evidence of gas vesicle formation under phase‐contrast imaging (D) compared to negative controls (E). Scale bar = 10 μm. [file BTM2-9-e10584-s001.pdf]

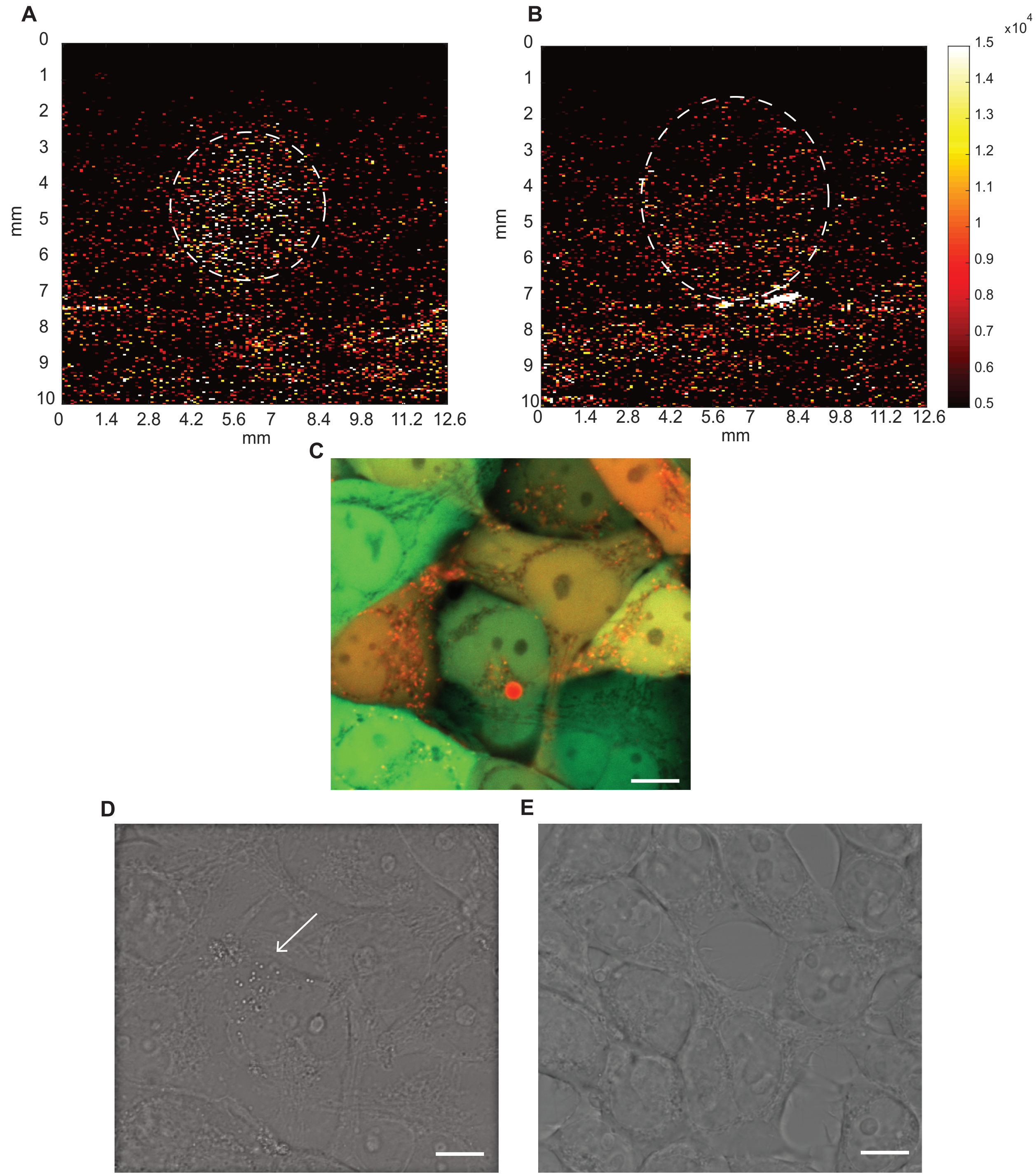

Figure S1. HEK293T $mARG_{ds}$ clonal cell line after freezing, thawing, and passaging for 5 passages post-thaw. Cells exhibit ultrasound contrast after doxycycline treatment (SNR = 24 dB, A) compared to cells that underwent no doxycycline treatment (SNR = 5.5 dB, B). Doxycycline-treated cells exhibit double-positive fluorescence (C) and evidence of gas vesicle formation under phase-contrast imaging (D) compared to negative controls (E). Scale bar = 10 µm.

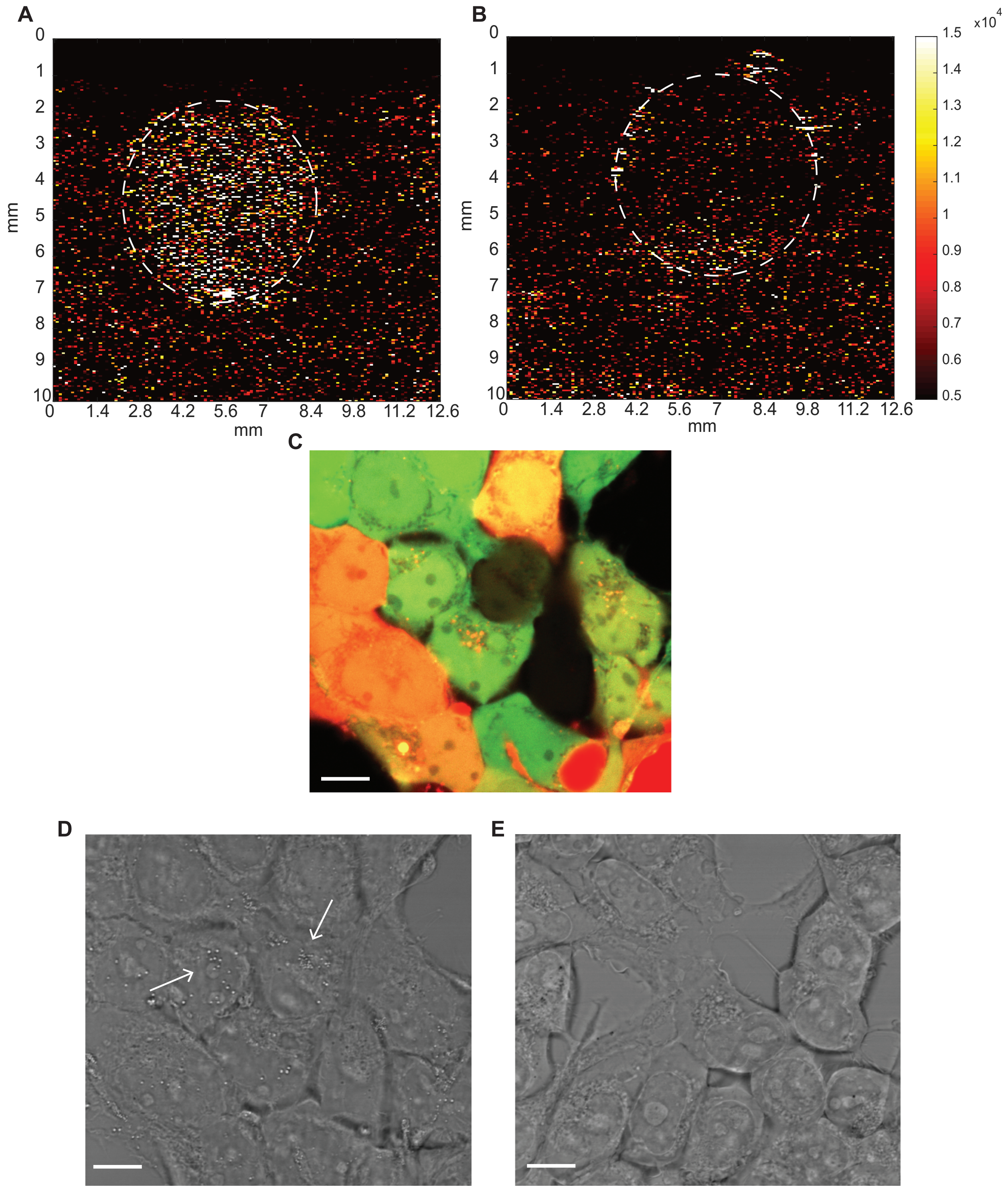

Figure S2. HEK293T $mARG_{ds}$ mixed-population cell line after freezing, thawing, and passaging for 5 passages post-thaw. Cells exhibit ultrasound contrast after doxycycline treatment (SNR = 29 dB, A) compared to cells that underwent no doxycycline treatment (SNR = 6.6 dB, B). Doxycycline-treated cells exhibit double-positive fluorescence (C) and evidence of gas vesicle formation under phase-contrast imaging (D) compared to negative controls (E). Scale bar = 10 µm.
