# Supplementary material for: A drug‐selectable acoustic reporter gene system for human cell ultrasound imaging
Source: Bioeng Transl Med. 2023 Aug 2;9(2):e10584. doi: 10.1002/btm2.10584 (PMC10905554; doi:10.1002/btm2.10584)
Supplement: Supplementary file 2 — Table S1. Primers used to PCR amplify mARG cassettes in order to clone into their respective drug‐resistant backbone via In‐Fusion cloning. [file BTM2-9-e10584-s002.docx]

**Supplementary Table 1:** Primers used to PCR amplify mARG cassettes in order to clone into their respective drug resistant backbone via In-Fusion cloning.

| **Primer Name** | **Sequence** |
| --- | --- |
| XLone-mARG1 Forward | cctaccctcgtaaaggtaccgccaccatgagcatccag |
| XLone-mARG1 Reverse | gaggtggtctactagttcacttgtacagctcgtccatg |
| XLone-mARG2 Forward | cctaccctcgtaaaggtaccgccaccatgaccgtgctg |
| XLone-mARG2 Reverse | gaggtggtctactagttcacttgtacagctcgtccatg |
| PB-UbC-mARG3 Forward | acggatccatgaattcgccaccatggccgtggaac |
| PB-UbC-mARG3 Reverse | tgcggccatgaagctttcacagcaggctgcccag |
